# Luminescent Lanthanide MOFs: A Unique Platform for Chemical Sensing

**DOI:** 10.3390/ma11040572

**Published:** 2018-04-07

**Authors:** Shu-Na Zhao, Guangbo Wang, Dirk Poelman, Pascal Van Der Voort

**Affiliations:** 1 Department of Chemistry, Center for Ordered Materials, Organometallics and Catalysis (COMOC), Ghent University, Krijgslaan 281 (S3), 9000 Gent, Belgium; shuna.zhao@Ugent.be (S.-N.Z.); Guangbo.Wang@UGent.be (G.W.); 2 LumiLab, Department of Solid State Sciences, Ghent University, Krijgslaan 281 (S1), 9000 Gent, Belgium; Dirk.Poelman@UGent.be

**Keywords:** metal–organic frameworks, lanthanide codoping, chemical sensors, ratiometric luminescence sensing

## Abstract

In recent years, lanthanide metal–organic frameworks (LnMOFs) have developed to be an interesting subclass of MOFs. The combination of the characteristic luminescent properties of Ln ions with the intriguing topological structures of MOFs opens up promising possibilities for the design of LnMOF-based chemical sensors. In this review, we present the most recent developments of LnMOFs as chemical sensors by briefly introducing the general luminescence features of LnMOFs, followed by a comprehensive investigation of the applications of LnMOF sensors for cations, anions, small molecules, nitroaromatic explosives, gases, vapors, pH, and temperature, as well as biomolecules.

## 1. Introduction

Metal–organic frameworks (MOFs) have attracted extensive attention over the past few decades. They are an emerging class of highly crystalline and porous materials formed by metal ions or metal clusters connected by multitopic organic linkers [1]. Their large surface areas, framework flexibility, and tunable pore surface properties, as well as “tailor-made” framework functionalities empower them to be promising candidates for a diverse range of applications, such as gas separation and sorption [2,3,4], luminescence [5,6], chemical catalysis [7,8], drug delivery [9], magnetism [10], chemical sensing [11,12,13], energy storage and conversion [14,15,16], proton conduction [17,18], and bio-imaging [19].

As a subclass of MOFs, luminescent MOFs possess potential for practical applications because of their explicit environments for luminophores in a crystalline state and characteristic optical performance [20]. Generally, the luminescent properties of MOFs generate from metal components and organic linkers with aromatic or conjugated π systems. The metal–ligand charge transfer (MLCT) related luminescence can extend their luminescence functionalities to another dimension. Moreover, some adsorbed guest molecules within MOFs are able to contribute to the luminescent properties. Until now, research on luminescent MOFs has mainly focused on the fundamental luminescent properties of MOFs, and the rational design of tunable luminescent MOFs for light emitting applications [21]. Recently, luminescent MOFs have been proven to be a unique platform for chemical sensing due to their special features, including (i) easily tunable luminescence that can be used as the appropriate sensing signal; (ii) specific functional groups (e.g., Lewis sites and open metal sites) that are able to promote preferred host–guest binding for selective sensing; and (iii) the permanent MOFs’ porosity that could concentrate the guest molecules, thereby enhancing detective sensitivity. Numerous luminescent MOF sensors have been developed and reported in the literature for detecting cations [22,23], anions [24,25], small molecules [26,27,28], biological molecules [29,30], explosive chemicals [31,32,33], vapors [34,35], and pH [36], as well as temperature [37,38,39].

Lanthanide MOFs (LnMOFs) have drawn much attention among the luminescent MOFs because of the unique luminescent properties of lanthanide ions, such as long lifetime, characteristic sharp emissions, large Stokes shifts, and high color purity with high quantum yields in the near-infrared and visible regions [40,41,42,43,44,45]. Additionally, the luminescent properties of lanthanide ions highly depend on the structural details of their coordination environment, offering a unique platform as chemical sensors. The combination of these characteristic luminescent properties of lanthanide ions with the intriguing topological structures of MOFs opens up promising possibilities for developing luminescent materials with special applications.

In this review, we present the most recent developments of LnMOFs as chemical sensors. We begin by briefly introducing the general luminescence features of LnMOFs, followed by a comprehensive investigation of the applications of LnMOF sensors with single or multiple luminescent centers. More specifically, LnMOF sensors for cations, anions, small molecules, nitroaromatic explosives, gases, vapors, pH, and temperature, as well as biomolecules will be discussed in detail in this review.

## 2. Luminescent Properties of LnMOFs

Generally, lanthanide ions (Ln^3+^) are characterized by successive filling of the 4f orbitals, with electronic configurations of [Xe]4f*^n^* (*n* = 0 to 14). These electronic configurations generate a rich variety of electronic levels with the number 14!/*n*!(14−*n*)!, resulting in interesting optical properties [46,47,48,49]. All of the Ln^3+^, except La^3+^ (4f^0^) and Lu^3+^ (4f^14^), exhibit luminescent f–f emissions, which almost cover the entire spectrum. Eu^3+^, Tb^3+^, Sm^3+^, and Tm^3+^ emit in the visible region with the color red, green, orange, and blue, respectively. Pr^3+^, Nd^3+^, Sm^3+^, Dy^3+^, Ho^3+^, Er^3+^, Tm^3+^, and Yb^3+^ show emissions the near-infrared region, while Ce^3+^ shows a broadband emission from 370 to 410 nm because of the 5d–4f transition [50].

**Figure 1:**
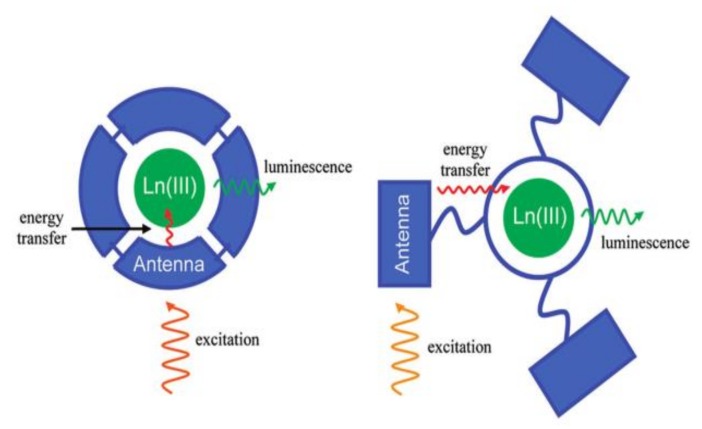
The antenna effect for lanthanide(III) (Ln(III)) sensitization, illustrated using the chromophoric chelate (right) and pendant chromophore (left) ligand designs. Reprinted with permission from [48]. Copyright 2009, American Chemical Society.

Typically, the 4f–4f transitions of Ln^3+^ are Laporte forbidden due to the 4f orbitals that are well-shielded by the filled 5s^2^5p^6^ subshells [51]. Consequently, direct photoexcitation of Ln^3+^ ions rarely produces highly luminescent materials due to the low absorption efficiency of the 4f–4f transitions. This problem can be overcome by the “antenna effect” (Figure 1), which commonly uses a strong absorbing chromophore to sensitize Ln^3+^ [52,53]. The overall process of antenna sensitization involves the following characteristic steps: (i) the organic ligands can absorb light upon excitation; (ii) the excitation energy is then transferred into Ln^3+^ excited states through intramolecular energy transfer; and (iii) Ln^3+^ ions undergo a radiative process by characteristic luminescence. This process could effectively increase the luminescence quantum yield of Ln^3+^ in normal conditions at room temperature. Furthermore, the solvent quenching and self-quenching of Ln^3+^ ions are almost nullified in LnMOFs due to the separation of Ln^3+^ ions by organic ligands. Consequently, LnMOFs exhibit strong luminescence and can be utilized as chemical sensors.

There are two other types of electronic transitions of Ln^3+^ ions: broad charge–transfer transitions (ligand–metal charge transfer (LMCT) and metal–ligand charge transfer (MLCT)) and broad 4f–5d transitions. They usually occur with high energies, resulting in rare observation in coordination compounds. However, the excitation energy of Sm^3+^, Eu^3+^, and Yb^3+^ can be transferred from an LMCT state to their 4f levels when the LMCT state lies at a high enough energy level. It is of great importance to investigate the numerous energy transfer processes for well-tuning the luminescent properties of LnMOFs.

The luminescence of Ln^3+^ ions is only possible from resonance levels, such as ^5^D_0_ for Eu^3+^, ^5^D_4_ for Tb^3+^, and ^2^F_5/2_ for Yb^3+^. The energies of resonance levels of Eu^3+^ (^5^D_0_), Tb^3+^ (^5^D_4_), and Yb^3+^ (^2^F_5/2_) lie at 17,250, 20,430, and 10,200 cm^−1^, respectively [54]. If the Ln^3+^ ions are excited to a nonresonance level, the excitation energy is dissipated through a nonradiative process until a resonance level is reached. Therefore, the lowest triplet state of the organic ligands in LnMOFs must be located at an energy level nearly equal to or above the resonance level of the Ln^3+^ ions. If the energy difference between the organic linkers and Ln^3+^ ions is too small, a thermally activated energy back-transfer will occur. On the other hand, large energy differences may lead to slower energy transfer rates. The energy of the triplet state must be elaborately tuned to maximize the transfer and minimize the back-transfer. Thus, the rational design of suitable organic ligands with the appropriate energy level is of great significance for the synthesis of LnMOFs with the desired luminescent properties.

## 3. LnMOFs for Chemical Sensing

LnMOFs have been widely studied in various sensor applications owing to their inherent porosity and the particular luminescent properties of Ln^3+^ ions. Most of the LnMOF sensors show luminescence intensity changes, including luminescence enhancement (turn-on response) and quenching (turn-off response) upon recognition of the analytes. Eu^3+^ and Tb^3+^ are commonly used as luminescent centers in LnMOF sensors because of their strong, characteristic red emission at around 614 nm and green emission at around 541 nm, respectively [55]. LnMOFs succeed in sensing ionic species, small molecules, explosive chemicals, and pH, as well as temperature. In addition, the inherent structural and chemical features of LnMOFs make them considerably useful in biosensing and bioimaging applications [56]. In the remainder of this section, recent developments of LnMOFs for chemical sensing will be discussed in detail.

### 3.1. LnMOFs for Cation Sensing

Sensing and detecting metal ions is of great significance in environmental and ecological systems. Some transition-metal cations, such as Cu^2+^, Fe^2+^, Fe^3+^, and Zn^2+^, are essential in biological metabolism. The excess or deficiency of these metal cations can cause various diseases, such as Alzheimer’s disease, Wilson’s disease, anemia, mental decline, etc. [57,58,59,60]. Hg^2+^, Pb^2+^, and Cd^2+^ are well-known toxic metal ions that can give rise to serious damage to the human body and environment [61,62]. Therefore, the design and preparation of efficient and straightforward metal ion probes are urgently needed.

**Figure 2:**
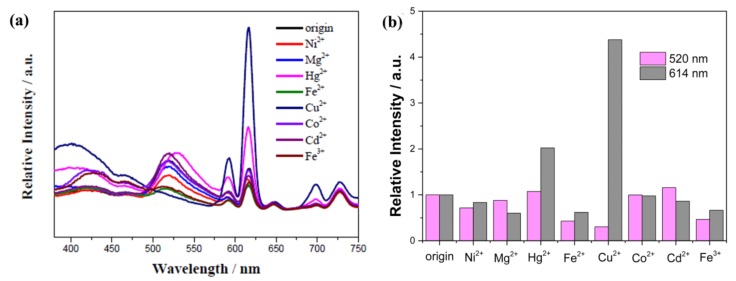
(**a**) PL spectra of FAM-ssDNA and Eu^3+^@Bio-MOF-1 dispersed into aqueous solutions of various metal ions with the concentration of 10^−5^ mol/L when excited at 323 nm; (**b**) Relative luminescence intensity of FAM at 520 nm and Eu^3+^ at 614 nm. Reprinted with permission from [69]. Copyright 2017 Elsevier B.V., New York, NY, USA.

In 2009, Chen et al. reported a new LnMOF [Eu(PDC)_1.5_(DMF)](DMF)_0.5_(H_2_O)_0.5_ (PDC = pyridine-3,5-dicarboxylate, DMF = N′N-dimethylformamide) with Lewis basic pyridyl sites for sensing Cu^2+^ ions [63]. The desolated MOF Eu(PDC)_1.5_ can selectively detect Co^2+^ and especially Cu^2+^ among other metal ions via a turn-off response. The authors hypothesized that the antenna efficiency of the PDC organic ligands was reduced by the binding of the pyridyl nitrogen atoms to Cu^2+^ or Co^2+^, resulting in luminescence quenching. From then on, many LnMOF sensors with unsaturated Lewis basic sites have been synthesized based on this mechanism for detecting metal ions [64,65,66,67,68]. Recently, Yan and coworkers developed a FAM-ssDNA and Eu^3+^@Bio-MOF-1 for sensing Cu^2+^ in aqueous solutions [69]. This luminescent hybrid material can simultaneously exhibit FAM and Eu^3+^ emissions by varying the ratio of Eu^3+^@Bio-MOF-1 and FAM-ssDNA. Cu^2+^ can quench FAM emission, while enhancing the luminescence intensity of Eu^3+^ (Figure 2). The mechanism behind this is possibly based on the interaction of Cu^2+^ and ssDNA.

Additionally, Fe^3+^ detection was achieved by Zheng et al. with [Eu(L_1_)(BPDC)_0.5_(NO_3_)]·H_3_O (H_2_L_1_ = 2,5-di(pyridin-4-yl)terephthalic acid, BPDC = biphenyl-4,4′-dicarboxylic acid) based on an excellent luminescence turn-off response with a remarkable detection limit (5 × 10^−7^ mol/L) over various other metal cations, including Na^+^, K^+^, Cu^2+^, Al^3+^, Mg^2+^, Cr^3+^, Zn^2+^, and Co^2+^ [70]. Sun and coworkers reported an anionic EuMOF, [H_2_N(CH_3_)_2_][Eu(H_2_O)_2_(BTMIPA)]·2H_2_O (H_4_BTMIPA = 5,5′-methylenebis(2,4,6-trimethylisophthalic acid)) with [H_2_N(CH_3_)_2_]^+^ cations in the tubular channels of the anionic frameworks, which exhibited luminescence quenching for Fe^3+^ and luminescence enhancement for Al^3+^ via ion-exchange between [H_2_N(CH_3_)_2_]^+^ cations and metal cations [71].

**Figure 3:**
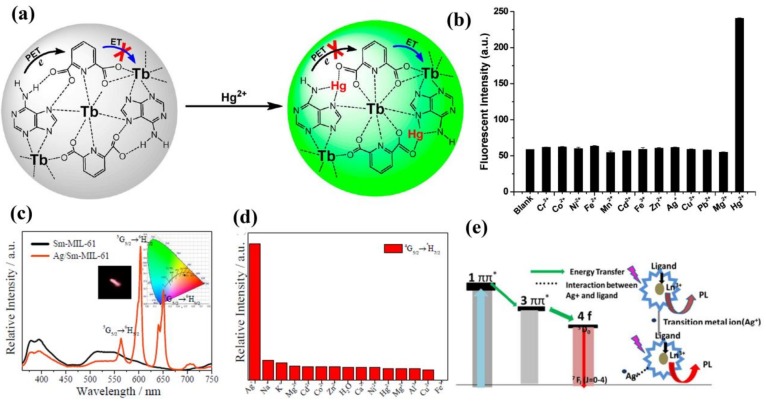
(**a**) Ad/Tb/DPA coordination polymer nanoparticles (CPNPs) for sensing of Hg^2+^ by photoinduced electron (PET) transfer; (**b**) Effect of various metal ions (1 μM) on the fluorescence intensity of Ad/Tb/DPA CPNPs at 545 nm. Reprinted with permission from [72]. Copyright 2012, American Chemical Society; (**c**) PL spectra of Sm-MIL-61 (black line) and Ag/Sm-MIL-61 (orange red line) when excited at 314 nm, the inset shows the CIE chromaticity diagram of Ag/Sm-MIL-61 (x: 0.4804 y: 0.3823) and corresponding photoluminescence color image under UV-] light irradiation at 314 nm; (**d**) The relative intensities of ^4^G_5/2_ → ^6^H_7/2_ at 603 nm upon Sm-MIL-61 in the presence of different metal ions; (**e**) Schematic illustration for the energy transfer from ligand to Ln^3+^ center. Reprinted with permission from [75]. Copyright 2017, The Royal Society of Chemistry.

Tan et al. prepared adenine-based lanthanide coordination polymer nanoparticles (CPNPs), consisting of adenine (Ad), a Tb^3+^ ion, and dipicolinic acid (DPA). It showed a turn-on luminescence response for Hg^2+^ in aqueous solutions [72]. Due to the photoinduced electron transfer (PET) process, the Ad can transfer energy to the DPA and simultaneously prevent intramolecular energy transfer from DPA to Tb^3+^, leading to the luminescence quenching of the CPNPs (Figure 3a). However, significantly enhanced luminescence (approximately fivefold) was observed in the CPNPs because of the suppression of the PET process from Ad to DPA by Hg^2+^, which was further confirmed by Fourier-transform infrared spectroscopy (FTIR) and lifetime study (Figure 3b). This Hg^2+^ nanosensor also showed superior selectivity and exceptionally high sensitivity up to the detection limit of 0.2 nM and can be used in biosensing and imaging. Li and coworkers reported a EuMOF ([Eu_2_(FDC)_3_DMA(H_2_O)_3_]·DMA·4.5H_2_O, H_2_FDC = 9,9-dimethyl-2,7-fluorenedicarboxylic acid,DMA = dimethylacetamide) for sensing Pb^2+^ in aqueous solutions through luminescence enhancement [73]. Another luminescence sensor for detecting Pb^2+^ based on a millimeter-sized TbMOF {[Tb(L_2_)(H_2_O)_5_]_n_⋅solvents H_2_L_2_**^−^** = 3, 5-dicarboxy-phenol anion ligand} was reported by Ji and coworkers [74]. It is the first high-efficiency MOF-based luminescence sensor for Pb^2+^ at a very low concentration and with the detection limit up to 10^−7^ M. A robust MOF, Sm-MIL-61(MIL-61 = Ga(OH)(btec)·0.5H_2_O, H_4_btec = Pyromellitic acid), was designed as an Ag^+^ sensor in aqueous solutions with high efficiency and selectivity (Figure 3c,d). The luminescence enhancement was due to a more efficient energy transfer from organic linkers to Sm^3+^ evoked by Ag^+^ (Figure 3e) [75].

### 3.2. LnMOFs for Anion Sensing

**Figure 4:**
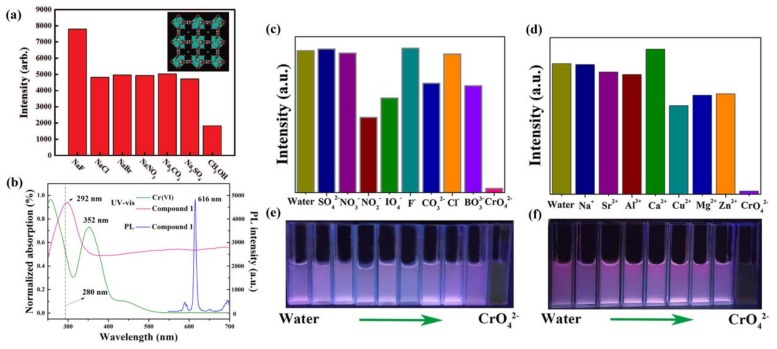
(**a**) ^5^D_4_ → ^7^F_5_ transition intensities of [Tb(BTC)·G] activated in different types of 10^−2^ M NaX and Na_2_X methanol solution (excited and monitored at 353 and 548 nm, respectively). The insert figure is the single crystal X-ray structure of [Tb(BTC)·G] activated in methanol containing NaF with the model of fluoride (green) at the center of the channel involving its hydrogen-bonding interaction with terminal methanol molecules (methanol oxygen, purple; the methyl group from methanol is omitted for clarity). Reprinted with permission from [78]. Copyright 2008, American Chemical Society. (**b**) Luminescence spectrum and UV−vis absorption spectra. (**c**,**d**) Luminescence intensity of ^5^D_0_ → ^7^F_2_ of Eu^3+^ at 616 nm dispersed in different aqueous solutions of various anions and cations. (**e**) Luminescence in different anion solutions (excited at 365 nm), corresponding to figure (**c**). (**f**) Luminescence in different cation solutions (excited at 365 nm), corresponding to figure (**d**). Reprinted with permission from [81]. Copyright 2017, American Chemical Society.

Various anions, such as halogen ions SO_4_^2−^, PO_4_^3−^, and CN^−^, are fundamental in environmental and biological systems [76]. Therefore, the sensing of such anions is a remarkably interesting topic to investigate. In recent years, LnMOF-based sensors have been successfully utilized for sensing inorganic anions [77,78,79,80]. Chen and coworkers synthesized a TbMOF [Tb(BTC)·G] (BTC = benzene-1,3,5- tricarboxylate, G = guest solvent) with OH groups in the terminal solvents [78]. This TbMOF showed a fourfold luminescence enhancement in the presence of F^−^, suggesting that this porous luminescent MOF is a promising candidate for sensing F^−^ (Figure 4a). The possible mechanism of luminescence enhancement by F^−^ ions lies in the stronger hydrogen bonding interactions between the F^−^ ion and the terminal methanol molecules that can restrict the stretching of the OH bond and thus reduce its quenching effect. The turn-off detection for F^−^ was achieved by Zhou and coworkers using an isostructural-doped LnMOF, [Eu_2x_Tb_2(1__−x)_(BPDC)(BDC)_2_(H_2_O)_2_]_n_ (H_2_BPDC = 2,2′-bipyridine-3,3′-dicarboxylic acid, H_2_BDC = 1,4-benzenedicarboxylic acid) [79]. The emission intensity of this codoped LnMOF reduced to almost zero in the presence of F^−^ in aqueous solutions, while the emission intensities showed no change in the presence of Cl^−^, Br^−^, or I^−^. The authors speculated that the F^−^ with smaller radii were trapped more easily in the MOF cavities than the other halogen anions, resulting in luminescence quenching. Shi et al. prepared two cationic hetero MOFs, [Ln_2_Zn(L_3_)_3_(H_2_O)_4_](NO_3_)_2_·12H_2_O_n_ (Ln = Eu and Tb, L_3_ = 4,4′-dicarboxylate-2,2′-dipyridine anion) for selective and reversible I^−^ detection in aqueous solutions [80]. I^−^ ions can quench the luminescence of these two cationic MOFs with a fast response time (10 s) and low detection limit (0.001 ppm). It is believed that I_3_^−^ ions are formed by the oxidation of I^−^ ions with the assistance of MOFs. They block the LMCT process by absorbing the excitation light, thus causing luminescence quenching.

Chromium is extensively used in various industrial processes causing Cr(VI) anions (CrO_4_^2−^ and Cr_2_O_7_^2−^) to often be present in all kinds of industrial wastewater It is one of the most prevalent, toxic heavy-metal ions of which excess intake can cause serious protein and DNA disruption, as well as damage the human enzyme system [82]. The detection of Cr(VI) anions was realized through a cationic EuMOF [Eu_7_(mtb)_5_(H_2_O)_16_]·NO_3_·8DMA·18H_2_O (H_4_mtb = 4-[tris(4-carboxyphenyl)methyl]benzoic acid) with a luminescence turn-off response [81]. The Cr(VI) anions can absorb the excitation light and hinder the energy absorption of the EuMOF, resulting in luminescence quenching (Figure 4b–f). This highly stable EuMOF sensor with excellent sensitivity and selectivity can also be utilized in real environmental conditions, such as lake water and sea water, suggesting the possible application of MOF chemical sensors in environmental fields. In another study, Li et al. synthesized a EuMOF [Eu(ipbp)_2_(H_2_O)_3_]·Br·6H_2_O (H_2_ipbpBr = 1-(3,5-dicarboxyphenyl)-4,4′-bipyridinium bromide) for the selective detection of Cr_2_O_7_^2−^ and CrO_4_^2−^ anions with K_sv_ of 8.98 × 10^3^ M^−1^ and 7.08 × 10^3^ M^−1^, respectively [83].

The commonly used strong oxidant, MnO_4_^−^, causes serious damage to the environment and human health. A stable luminescence sensor for MnO_4_^−^ was designed by Yan and coworkers using an In-MOF supporter encapsulated with Eu^3+^ ions [24]. Upon the addition of MnO_4_^−^, the luminescence of In-MOF-Eu was quenched to dark, corresponding to the competition of MnO_4_^−^ with the organic linkers for absorption of excitation light. Moreover, the color of the MOF-based fluorescence test paper can be observed to go from red to black by the naked eye under UV light irradiation when immersed in different MnO_4_^−^ concentrations. Li and coworkers utilized a heterometallic alkaline earth–lanthanide MOF {[Ba_3_La_0.5_(*μ*_3_-L)_2.5_(H_2_O)_3_(DMF)]·(3DMF)}_n_ (H_3_L_4_ = p-terphenyl-3,4″,5-tricarboxylic acid) to detect MnO_4_^−^ with significant quenching over other anions, such as PO_4_^3^^−^, Cl^−^, SiF_6_^2^^−^, CO_3_^2^^−^, HCO_3_^−^, BF_4_^−^, NO_3_^−^, Ac^−^, SCN^−^, SO_4_^2^^−^, Br^−^, I^−^, F^−^, IO_3_^−^, BrO_3_^−^, and ClO_4_^2^^−^ [84]. This probe exhibited high selectivity and sensitivity for MnO_4_^−^ ions with high quench efficiency constants K_sv_ = 7.73 × 10^3^ M^−1^, as well as a low fluorescence-detection limit (0.28 μM (S/N = 3)).

**Figure 5:**
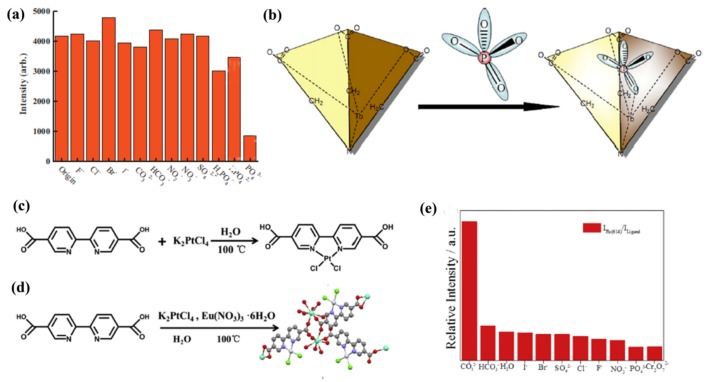
(**a**) Comparison of the ^5^D_4_ → ^7^F_5_ transition intensities of TbNTA solid activated in 10^−2^ M NaX aqueous solution. (**b**) A schematic representation of the phosphate anion sensor. Reprinted with permission from [86]. Copyright 2010 Elsevier B.V. (**c**,**d**) Schematic illustration of the synthetic process of Eu/Pt-MOFs. The Eu, C, O, Pt, N, and Cl atoms are represented by blue, grey, red, white, purple and green, respectively. Hydrogen atoms and uncoordinated water molecules are excluded for clarity. (For interpretation of the references to color in this figure legend, the reader is referred to the web version of this article.) (**e**) A histogram demonstrating the value of *I*_Eu(614)_/*I*_Ligand_ according to the fluorescence spectrum. Reprinted with permission from [25]. Copyright 2017 Elsevier Ltd., Toronto, Canada.

PO_4_^3−^ ions are also a type of pollutant anion that can cause water eutrophication and serious pollution in aquatic ecosystems [85]. A luminescent TbMOF TbNTA·H_2_O (NTA = nitrilotriacetate) for sensing PO_4_^3−^ ions is provided by Qian and coworkers [86]. The luminescence intensity of TbNTA·H_2_O quenched significantly in the presence of PO_4_^3−^, while it showed almost no change upon exposure to F^−^, Cl^−^, Br^−^, I^−^, NO_3_^−^, NO_2_^−^, HCO_3_^−^, CO_2_^2^^−^, or SO_4_^2^^−^ (Figure 5a). They further discussed possible sensing mechanisms based on the matching degree of TbNTA·H_2_O with different anions. The Tb–O bond may dilute the energy that transferred to Tb^3+^ via non-radioactive relaxation after incorporating PO_4_^3–^ into TbNTA·H_2_O (Figure 5b). Another PO_4_^3–^ probe was achieved by Zhao and coworkers using a regenerable EuMOF {[Eu_1.5_(BTB)_1.5_(H_2_O)]·3DMF}_n_ (H_3_BTB = 1,3,5-benzenetribenzoate) [87]. The recyclable performance of this EuMOF was investigated by fast and simple methods. Generally, this EuMOF was immersed in an PO_4_^3−^ aqueous solution (10^−3^ M) for 20 s to completely form EuMOF-PO_4_^3−^, then EuMOF-PO_4_^3−^ was washed in water several times to obtain the original EuMOF. The results demonstrate the promising practical applications of this recyclable PO_4_^3–^ probe. 

Recently, Yan and coworkers reported a heterobimetallic Eu/Pt-MOF with dual emissions from both organic linkers and Eu^3+^ that exhibited facile, fast, and ratiometric detection of CO_3_^2−^ (Figure 5c,d) [25]. The authors posited that the interaction with CO_3_^2^^−^ suppressed the ligand-centered luminescence and enhanced the luminescence of Eu^3+^, resulting in the maximum intensities ratio of Eu^3+^ (614 nm) to ligand (Figure 5e). The results indicate that the ratiometric sensing methodology could be an efficient platform for analytical monitoring of trace CO_3_^2^^−^ in real samples due to the excellent orientation selectivity of CO_3_^2^^−^.

### 3.3. LnMOFs for Small Molecule Sensing

**Figure 6:**
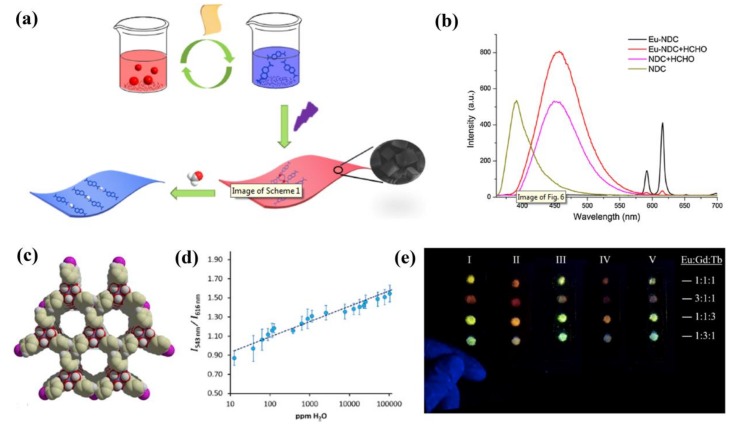
(**a**) Schematic representation of the synthesis process of Eu-NDC@HPAN and the luminescence quenching phenomenon of formaldehyde (HCHO) to Eu-NDC@HPAN; (**b**) Luminescence spectra of deprotonated NDC and Eu-NDC (0.25 mg/mL) (before and after treatment with formaldehyde) (λ_ex_ = 360 nm). Reprinted with permission from [90]. Copyright 2017 Elsevier B.V.; (**c**) Space-filling view along the c axis shows 1D hexagonal channels of PCM-22; (**d**) Relative photoemission response ratios obtained upon the addition of trace H_2_O to Eu_1_:Tb_5_-PCM-22 presoaked in D_2_O show a linear response. Error bars were obtained from three separate experiments; (**e**) Model dipstick detectors demonstrated for H_2_O sensing: (I) as-synthesized, (II) desolvated in air with a heat gun, (III) after exposure to H_2_O, (IV) reactivation using a heat gun, and (V) re-exposure to H_2_O. Reprinted with permission from [91]. Copyright 2017 Elsevier Inc.

Formaldehyde (HCHO) is widespread in construction, furniture, and particle board, posing an impact on human health, such as watery eyes, asthma, and respiratory irritation [88]. Yu and coworkers developed a ratiometric luminescence HCHO probe through incorporation of Eu^3+^ ions into NH_2_-UiO-66 under microwave irradiation conditions [89]. The dual-emitting luminescence originated from the characteristic red emission of Eu^3+^ ions (615 nm) and linker-to-cluster (Eu-oxo or Zr-oxo) charge transfer transition-related emission (465 nm). The interaction of the free amino groups with HCHO can drastically enhance emission around 465 nm due to the added electron transfer from the amino group with lone pair electrons to the positively charged HCHO. This is in contrast to the emission of Eu^3+^ at 615 nm that was only slightly enhanced. Then, a ratiometric luminescence HCHO probe was performed based on the intensity ratio of two emission bands at 465 nm and 615 nm. The results indicated that the fabrication of a ratiometric luminescence probe based on multiband luminescent MOFs can serve as a common sensing method for organic molecules. Another ratiometric luminescence sensor for HCHO was reported by Yang and coworkers [90]. This self-calibrating luminescent film was fabricated directly by growing Eu-NDC (H_2_NDC = 2,6-naphthalenedicarboxylate) on hydrolyzed polyacrylonitrile (HPAN) via a layer-by-layer strategy (Figure 6a). The Eu-NDC@HPAN thin film can detect HCHO via a ratiometric luminescence approach with a 3.2-fold increase of the relative ratio of luminescence intensities at 453 nm and616 nm. It has been proposed that the Eu-NDC frameworks will decompose after adding HCHO, while the NDC ligands regenerate, resulting in luminescence quenching and enhancing of Eu^3+^ ions and NDC, respectively (Figure 6b). The remarkable selectivity, sensitivity, and water stability of this film HCHO probe indicates its potential use in life sciences.

Recently, Humphrey and coworkers reported a rare example of a LnMOF probe for detecting trace H_2_O in D_2_O [91]. D_2_O is an isotopically labeled version of H_2_O and is widely used in chemical analysis and medicine [92]. High-purity D_2_O is essential in various spectroscopic and synthetic applications. The codoped PCM-22 [Ln(tctp)(OH_2_)_3_]·3(1,4-dioxane) (Ln = Eu^3+^, Tb^3+^ and Gd^3+^, tctpH_3_ = tris(p-carboxylato)triphenylphosphine (P(C_6_H_4_-*p*-CO_2_H)_3_)) has a 3D structure consisting of puckered 2D honeycomb sheets with large hexagonal channels and exhibits the characteristic luminescence of Eu^3+^ and Tb^3+^ (Figure 6c). This material allows for immediate solvent identification through color changes, which can easily be observed by the naked eye. Interestingly, the sensor can also be employed to quantitatively detect trace H_2_O in D_2_O (Figure 6d,e), as well as acetone, ethanol, and acetonitrile by uncomplicated spectrophotometry. To the best of our knowledge, this codoped LnMOF is the first material-based sensor for detecting H_2_O in D_2_O from 10 to 120,000 ppm. Buschbaum et al. proposed a new approach to obtain a ratiometric H_2_O probe by using superparamagnetic microparticles Fe_3_O_4_/SiO_2_ as a core and different LnMOFs as a shell [93]. [Eu_2_(BDC)_3_]·2H_2_O·2DMF (BDC^2−^ = benzene dicarboxylate) and [Ln_2_Cl_6_(bipy)_3_]·2bipy (Ln = Eu and Tb; bipy = 4,4′-bipyridine) were chosen to functionalize the Fe_3_O_4_/SiO_2_ core, forming a color-tuned yellow-emitting Fe_3_O_4_/SiO_2_@mixed-MOF composite system. The luminescence of two MOFs decreased unequally upon the presence of H_2_O, allowing for a quantitative detection of H_2_O content by the Tb^3+^- and Eu^3+^-based luminescence intensity ratio. In addition, the Fe_3_O_4_/SiO_2_@mixed-MOF composite system can easily be removed from the liquid phase by means of a magnet. 

**Figure 7:**
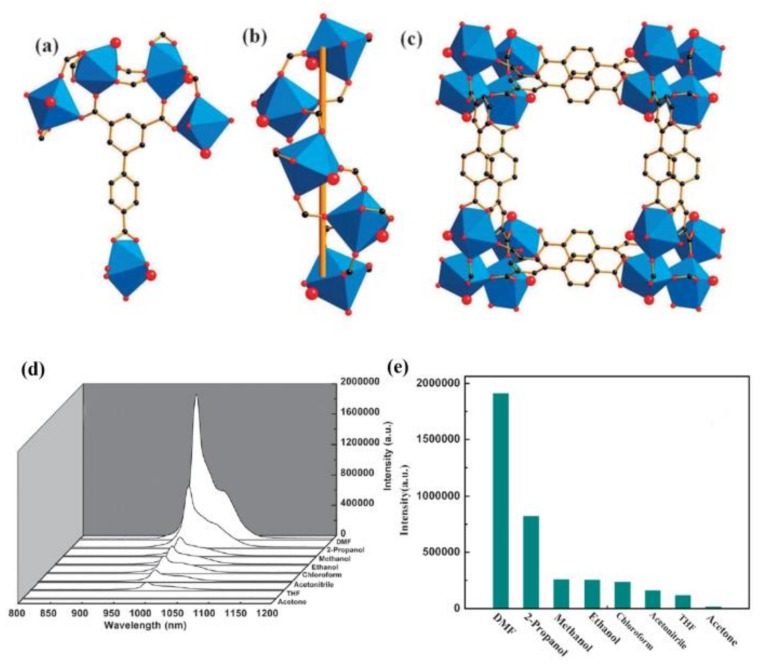
Crystal structure of YbMOF indicating (**a**) the BPT tricarboxylate linker and coordination environments of all Yb atoms related by symmetry; (**b**) 1D helical rod [Yb(CO_2_)_3_]*_n_* as the infinite SBU; and (**c**) 1D micropore of about 7.2 × 7.2 Å along c axis (Yb, blue polyhedra; C, black; O, red: terminal water molecules, large red sphere); (**d**) the PL spectra; and (**e**) the ^2^F_5/2_–^2^F_7/2_ transition intensities of YbMOF introduced into various pure solvent emulsions when excited at 304 nm. Reprinted with permission from [94]. Copyright 2011 The Royal Society of Chemistry.

A EuMOF [Eu(FBPT)(H_2_O)(DMF)] (FBPT = 2′-fluoro-biphenyl-3,4′,5-tricarboxylate) for sensing acetone was reported by Zhang and coworkers [22]. The luminescence intensity of this EuMOF primarily depends on the organic solvents, particularly in the case of acetone, which exhibited the most significant quenching effect. It has been suggested that the competition of absorbing excited light energy between FBPT and acetone plays an important role in their luminescence diminishment. Guo et al. examined the capability of NIR luminescent YbMOF Yb(BPT)(H_2_O)·(DMF)_1.5_(H_2_O)_1.25_ (BPT = biphenyl-3,4′,5-tricarboxylate) for organic molecule sensing (Figure 7a–c) [94]. When excited at 326 nm, the active Yb(BPT) exhibits typical NIR emission of Yb^3+^ ions at 980 nm (Figure 7d). The NIR emission showed significant quenching and enhancement effects in the presence of acetone and DMF, respectively (Figure 7e). This study opens up a new approach for luminescent MOF-based sensors with NIR emission, demonstrating their potential applications in biological systems. Liu and coworkers synthesized a heterometallic MOF, {[Tb_2_(ODA)_6_Cd_3_(H_2_O)_6_]·6H_2_O}_n_ (ODA = oxydiacetic acid), that can selectively detect ethanol and 2-propanol with luminescence turn-on and turn-off responses, respectively [95]. A series of MOFs composed of 4,4′-oxybis(benzoate) (OBA) ligands and suitable cations were reported by Hus and coworkers [96]. The MOF Na[Tb(OBA)_2_]_3_·0.4DMF_3_·1.5H_2_O shows the strongest emission in the presence of BuOH and EtOH, whereas a much weaker emission was found in the presence of MeOH and H_2_O. One possible mechanism explaining this is that MeOH and H_2_O are trapped in the MOF cavities entering the coordination spheres of Tb^3+^. This potentially causes the luminescence quenching effect. The EtOH and BuOH molecules then protect the Tb^3+^ from quenching by O–H oscillators because of the relatively sterical bulky alkyl groups. Wang et al. reported a codoped LnMOF, [LnL_5_(H_2_O)_2_]·2H_2_O (Ln = Eu and Tb, H_3_L_5_ = 4-(2-carboxyphenoxy)benzene-1,3-dioic acid) showing good sensitivity to CH_3_CN and nitrobenzene [97]. The emission can be enhanced remarkably in the presence of CH_3_CN, while nitrobenzene can significantly quench the emission.

Benzene and its homologues, a prime type of toxic pollutant, bring great harm to both the environment and humans. It is therefore of significant importance to develop an efficient and easily processed approach to detect this kind of pollutant. Cheng and coworkers constructed a red luminescence sensor based on {[Eu_2_(L_6_)_3_(DMF)_2_]·DMF·MeOH}*_n_*(H_2_L_6_ = 5-(4*H*-1,2,4-triazol-4-yl)benzene-1,3-dicarboxylic acid) to effectively detect polychlorinated benzenes [98]. This EuMOF represents a highly efficient quenching effect on detecting polychloriznated benzenes, including 1,2,4-trichlorobenzene, 1,2,3,4-tetrachlorobenzene, 1,2,4,5-tetrachlorobenzene, pentachlorobenzene, and hexachlorobenzene, which can be ascribed to the competition of the absorption of the excitation light between the analytes and ligands. Weng et al. fabricated a dual-emissive hybrid N-GQDs/Eu^3+^@Mg-MOF (N-GQDs = N atom-doped graphene quantum dot, Mg-MOF = {[Mg_3_(ndc)_2.5_(HCO_2_)_2_(H_2_O)][NH_2_Me_2_]⋅2H_2_O⋅DMF} 1,4-ndc = 1,4-naphthalenedicarboxylate) and employed it as a ratiometric luminescence sensor for decoding benzene homologues [53]. It exhibits dual-emission of N-GQDs and Eu^3+^ when excited at 394 nm, while the emission of the ligands and Eu^3+^ can be collected when excited at 349 nm. Thus, a 2D decoded map with *I*_L_/*I*_Eu_ as abscissa and *I*_Eu_/*I*_N-GQDs_ as ordinate is established to identify benzene homologues. The results demonstrated that the decoded map can be used for the precise recognition of unknown compounds.

### 3.4. LnMOFs for Nitroaromatic Explosive Sensing

It is of great importance to selectively and rapidly detect nitroaromatic explosives in environmental monitoring, civilian safety, and homeland security [99]. The current methods for explosive detection are limited by their equipment demands and cost drawbacks [100]. However, luminescence sensing has proven to be an excellent detection technique for explosives owing to its speed and cost effectiveness, as well as to the fact that it is easily portable [101]. 

**Figure 8:**
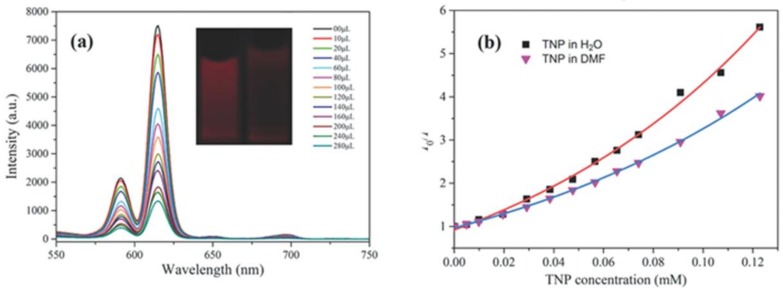
(**a**) Effect on the emission spectra of the activated EuMOF dispersed in H_2_O upon incremental addition of a 2,4,6-trinitrophenol (TNP) aqueous solution (1 mM) (λ_ex_ = 362 nm). The legend indicates the overall concentration of TNP. Inset: A photograph showing the original fluorescence (**left**) and the fluorescence quenching (**right**) upon the addition of 280 μL TNP (UV light, 365 nm). (**b**) Stern–Volmer plots of *I*_0_/*I* versus the TNP concentration in DMF and water. Reproduced with permission from [107]. Copyright 2014 Wiley-VCH.

The detection of explosives using LnMOF-based luminescence sensors is usually performed in a turn-off manner. The luminescence quenching effect can be assigned to the photoinduced electron or energy transfer. The conduction band (CB) of the electron-rich MOF lies higher than the lowest unoccupied molecular orbitals (LUMOs) energy of the electron-deficient analytes. This allows for the electron transfer from the CB of the MOF sensors to the LUMOs of nitro analytes causing luminescence quenching [102]. Another possible mechanism for luminescence quenching is the competition of the absorption of the excitation light energy between the MOF ligands and nitro analytes. Based on these two possible sensing mechanisms, great success has been reported for sensing nitroaromatic explosives, such as nitrobenzene (NB), m-nitrotoluene (m-NT), o-nitrotoluene (o-NT), 3-nitrophenol (3-NP), 4-nitrophenol (4-NP), 2,4-dinitrophenol (2,4-NP), 2,4,6-trinitrophenol (TNP), and 2,4,6-trinitrotoluene (TNT) (Figure 8) [103,104,105,106,107].

The luminescence quenching efficiency of LnMOFs towards nitroaromatic explosives was analyzed using a quenching constant *K_sv_* (M^−1^) and detection limits. The quenching constant *K_sv_* (M^−1^) is calculated by using the Stern–Volmer (SV) equation, (*I*_0_/*I*) = *K*_sv_[A] + 1, where *I*_0_ and *I* are the luminescence intensities before and after the addition of the analyte, respectively, and where [A] is the molar concentration of the analyte. The detection limit was calculated by *K*_sv_ values and the standard deviation (*S*_b_), defined as n*S*_b_/*K*_sv_ [108].

### 3.5. LnMOFs for Gas and Vapor Sensing

**Figure 9:**
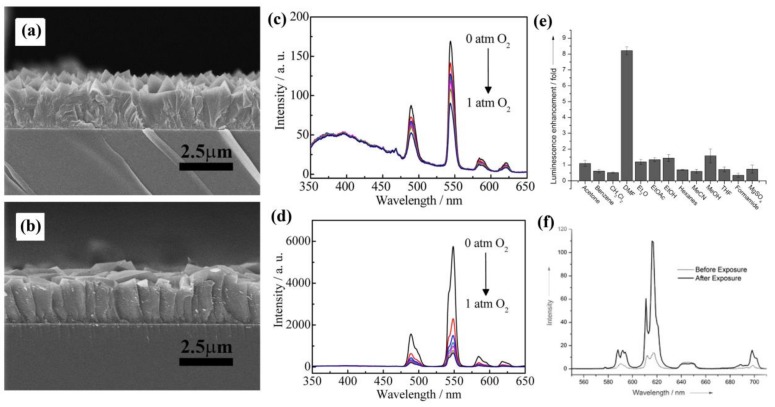
SEM images of CPM-5⊃Tb^3+^ (**a**) and MIL-100(In)⊃Tb^3+^ (**b**) films. Emission spectra of activated (**c**) CPM-5⊃Tb^3+^ and (**d**) MIL-100(In)⊃Tb^3+^ films under different oxygen partial pressure (Po_2_). Reproduced with permission from [109]. Copyright 2014 American Chemical Society. (**e**) Increase in Eu emission intensity of [Eu_2_L_3_(H_2_O)_4_]·3DMF (*I*_after_/*I*_before_-1) after incubation for 24 h under various solvent vapors and with anhydrous MgSO_4_. The intensity is measured at 616 nm. Error bars indicate the standard deviations of three or four parallel experiments. (**f**) Emission spectra of [Eu_2_L_3_(H_2_O)_4_]·3DMF before and after exposure to DMF vapor (excitation at 323 nm). The broad peak around 640 nm arises from scattering. Reproduced with permission from [110]. Copyright 2013 Wiley-VCH, Weinheim, Germany.

The luminescent MOF films, CPM-5⊃Tb^3+^ and MIL-100(In)⊃Tb^3+^, were designed by Qian and coworkers as a fast-response oxygen probe (Figure 9a,b) [109]. The luminescence intensities of the activated CPM-5⊃Tb^3+^ and MIL-100(In)⊃Tb^3+^ decreased gradually with increasing O_2_ pressure. MIL-100(In)⊃Tb^3+^ showed higher quenching efficiencies (88%) than did CPM-5⊃Tb^3+^ (47%) at 1 atm of O_2_ (Figure 9c,d). This is because the exposed carboxylate acids in MIL-100(In) can form Tb–O bonds with Tb^3+^ ions, leading to the intramolecular energy transfer, whilst Tb^3+^ merely balances cations in the pores of CPM-5, leading to intermolecular energy transfer. The high-oxygen sensitivity and short response/recovery time of MIL-100(In)⊃Tb^3+^ indicate their potential in sensing gases or vapors.

Song and coworkers exploited a EuMOF [Eu_2_(L_7_]_3_(H_2_O)_4_]·3DMF (L_7_ = 2′,5′-bis(methoxymethyl)-[1,1′:4′,1″-terphenyl]-4,4″-dicarboxylate) for sensing DMF vapor with a turn-on response [110]. A water-exchanged framework was formed by submerging the EuMOF in distilled water for 3 days and consequently showed much weaker Eu^3+^-based emission due to the quenching effect of the water molecules. The Eu^3+^ luminescence intensity exhibits a more than eightfold increase in the presence of DMF vapor. This is primarily due to partial replacement of the channel water by DMF molecules that reduce the quenching effect of the water molecules. This explanation was further confirmed by the fluorescence decay of deuteroxide- and water-exchanged samples. Moreover, DMF molecules within the channels of the compound can also modulate the energy levels of the ligands, thus promoting the LMCT process, all confirmed by NMR and XRD studies.

**Figure 10:**
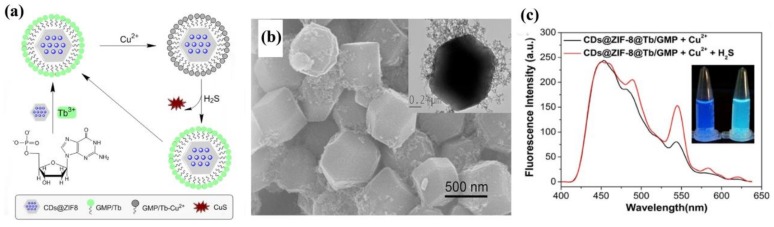
(**a**) Schematic illustration of the preparation of CDs@ZIF-8@GMP/Tb and its working principle for ratiometric detection of H_2_S; (**b**) SEM images of CDs@ZIF-8@GMP/Tb. Inset is the corresponding TEM image; (**c**) Emission spectra of CDs@ZIF-8@Tb/GMP in the presence of Cu^2+^ and Cu^2+^ + H_2_S. Reproduced with permission from [112]. Copyright 2017 Elsevier B.V., New York, NY, USA.

Besides the distinct rotten egg smell for which this toxic gas is commonly known, hydrogen sulfide (H_2_S) is of great importance in biological systems, as well as the cause of acid rain and other environmental problems [111]. Tan and coauthors developed a ratiometric sensor for H_2_S based on Cu^2+^-mediated fluorescence of LnCPs doped with carbon dots (CDs) (CDs@ZIF-8@GMP/Tb) [112]. GMP/Tb on the surface of ZIF-8 (zeolitic imidazolate framework-8) displays a typical ON-OFF-ON behavior upon the sequential addition of Cu^2+^ and H_2_S, an observation that can be put to use in response signaling (Figure 10a,b). The fluorescence of the CDs of CDs@ZIF-8@GMP/Tb remains unchanged in the presence of Cu^2+^ or/and H_2_S, empowering CDs to be of good reference. As a result, a ratiometric fluorescence sensor based on CDs@ZIF-8@GMP/Tb for sensing H_2_S was fabricated (Figure 10c). The high selectivity towards H_2_S against other anions (e.g., thiols and biological species) and the distinct feature of reversible sensing of this ratiometric sensor will promote the development of more sensitive ratiometric sensors based on LnMOFs. Another H_2_S probe was reported by Yang and coworkers based on the postsynthetic modification of Tb^3+^@Cu-MOF [113]. The Tb^3+^@Cu-MOF (Cu-MOF: [Cu(HCPOC)_2_]_n_ H_2_CPOC = 5-(4′-carboxylphenoxy) nicotinic acid) exhibits a typically weak emission of Tb^3+^ yet a strong ligand-centered emission. The Tb^3+^-based emission can be strongly enhanced by H_2_S due to its superior affinity towards Cu^2+^ ions. The detection performance of Tb^3+^@Cu-MOF (1.20 μM) is capable of meeting that of biological systems indicating its potential in real-time organismal H_2_S sensing.

Tanase and coworkers reported a dual-mode humidity sensor based on a EuMOF [Eu(H_2_O)_2_(mpca)_2_Eu(H_2_O)_6_W(CN)_8_]⋅*n*H_2_O (mpca = 2-pyrazine-5-methyl-carboxylate) [114]. This EuMOF has a robust three-dimensional network with significant hydrophilic open channels filled with water molecules. The luminescence intensity of the EuMOF gradually decreases as the humidity increases. This effective and remarkably reliable humidity sensor also shows good linearity over a broad humidity range from 0% to 100% RH. Moreover, this sensing material was also examined for electrical detection methods. The recovery time of these methods was found to be similar to that in the photoluminescence measurement.

### 3.6. LnMOFs for pH Sensing

**Figure 11:**
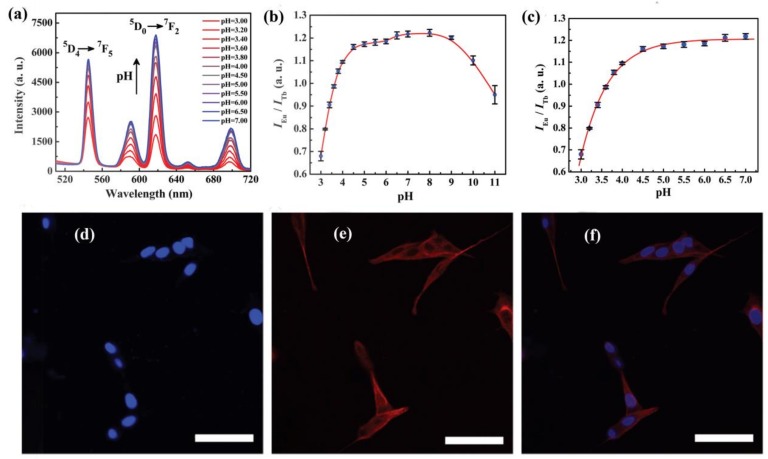
(**a**) pH-dependent emission spectra of Eu_0.034_Tb_0.966_-NMOF (W = 20, W is defined as the water-to-surfactant molar ratio) in the pH ranging from 3.00 to 7.00; (**b**) pH-dependent intensity ratio of Eu^3+^ (618 nm) to Tb^3+^ (545 nm) in the pH ranging from 3.00 to 11.00; (**c**) The fitted curve of Eu_0.034_Tb_0.966_-NMOF (W = 20) in the pH ranging from 3.00 to 7.00; (**d**,**e**) fluorescence and (**f**) overlapped confocal microscopy images of fixed PC12 cells incubated with 50 μg·mL^−1^ Eu_0.034_Tb_0.966_-NMOF for 24 h. Microtubular cytoskeleton (tubulin, red) and nuclei (blue) were fluorescently stained. The scale bar is 50 μm. Reproduced with permission from [120]. Copyright 2017 The Royal Society of Chemistry.

The need to explore fast pH sensing in industry, biomedicine, and many other environmental fields in order to monitor pH values and changes in biological systems and living cells has recently become of top priority [115]. The advantages of luminescence-based pH probes including quick response and high sensitivity, as well as easy operation, making them particularly desirable [116]. Chen and coworkers designed a pH-sensitive MOF nanoparticle using DMF and 1,10-phenanthroline (Phen) as ligands with Tb^3+^ ions based on the intramolecular-charge-transfer (ICT) effect [117]. A DMF molecule contains both an electron-donor and -acceptor part, allowing it to generate ICT [118]. It can furthermore change the Tb^3+^-based luminescence through the antenna effect. Consequently, the protonation of H^+^ could change the charge transfer of DMF and further change the antenna effect for Tb^3+^, in turn resulting in a change of Tb^3+^-based luminescence. The Phen molecule in the nanoparticle was used to improve such a change and reduce the luminescence quenching effect of Tb^3+^ by replacing the coordinated water molecules. The emission intensity of DMF–Tb was improved approximately 4 times, while the emission intensity of DMF–Tb–Phen was improved 10 times due to a decrease of the ICT effect and increase of the antenna effect on the Tb^3+^ ions upon adding H^+^. This MOF nanoparticle pH sensor with high specificity and sensitivity could be used in strong acidic conditions, indicating its potential applications in biological systems. Qian and coworkers fabricated a fluorescence pH sensor by encapsulating Eu^3+^ ions into the pores of the nanoscale UiO-67-bpydc (bpydc = 2,2′-bipyridine-5,5′-dicarboxylic acid) [119]. The luminescence intensity of Eu^3+^@UiO-67-bpydc shows a significant luminescence turn-off response in acidic solutions while exhibiting florescence enhancement in basic solutions. This is because protonation and deprotonation of the ligands first change the excited-state energy level of the ligands followed by a change in ligand-to-Eu energy transfer efficiency, explaining the different changes in the Eu^3+^-based luminescence. This Eu^3+^@UiO-67-bpydc pH sensor is stable within a wide pH range of 1.06 to 10.99 and can thus be used in physiological environments (pH = 6.80–7.60). The bio-compatibility of Eu^3+^@UiO-67-bpydc was further confirmed by an MTT (MTT = 3-(4,5-dimethylthiazol-2-yl)-2,5-diphenyl-tetrazolium bromide) assay. Cell imaging results demonstrate that the Eu^3+^@UiO-67-bpydc pH probe could be a promising candidate for monitoring pH both in vitro and in vivo. Very recently, the same group reported another luminescence pH sensor based on a nanoscale mixed LnMOF Eu_0.034_Tb_0.966_(fum)_2_(ox)(H_2_O)_4_ (fum = fumarate, ox = oxalate) [120]. The Eu_0.034_Tb_0.966_(fum)_2_(ox)(H_2_O)_4_ pH sensor shows high stability in aqueous solutions. Moreover, its morphology and size can easily be adjusted by changing the amount of CTAB surfactant. The mixed LnMOF exhibits both Tb^3+^ (545 nm) and Eu^3+^ (618 nm) emissions, which can be used for sensing pH values ranging from 3.00 to 7.00 in a ratiometric manner (Figure 11a–c). The MTT analysis and optical microscopy assay show that this mixed LnMOF sensor has low cytotoxicity and favorable biocompatibility (Figure 11e–f), indicating its potential to be applied as a pH sensor in physiological environments.

### 3.7. LnMOFs for Temperature Sensing

Temperature is an important thermodynamic parameter in human life and scientific investigations. Therefore, accurate temperature measurement is essential in both scientific and human development. Among the approaches for temperature determination, luminescence-based measurements have achieved tremendous attention with regards to their prominent advantages, including noninvasiveness, fast response, accuracy, high spatial resolution, and ability to work in strong electro or magnetic fields [121]. However, the most luminescent thermometers depend on a single emission susceptible to errors because of sample concentration changes and drifts of the optoelectronic system.

Qian and coworkers fabricated the first self-calibrated luminescent temperature sensor using a mixed LnMOF Eu_0.0069_Tb_0.9931_-DMBDC (DMBDC = 2,5-dimethoxy-1,4-benzenedicarboxylate) [37]. For Tb-DMBDC and Eu-DMBDC, the characteristic luminescence gradually decreases because of thermal activation of nonradiative decay pathways. However, Eu_0.0069_Tb_0.9931_-DMBDC exhibits a significant temperature-dependent luminescent behavior as the temperature increases from 10 to 300 K. The Tb^3+^-based emission in Eu_0.0069_Tb_0.9931_-DMBDC decreases as the temperature increases, while that of the Eu^3+^ ions increases. This can be ascribed to the efficient energy transfer from Tb^3+^ to Eu^3+^ based on the phonon-assisted Förster transfer mechanism, an effect confirmed by luminescence lifetime measurements. The good linear relationship between the *I*_Tb_/*I*_Eu_ ratio and temperature in the range of 50–200 K suggests that Eu_0.0069_Tb_0.9931_-DMBDC is an excellent temperature thermometer within this temperature range. These results suggest that mixed LnMOFs featuring temperature-dependent luminescence can be ideal candidates for self-referencing temperature sensing. Since then, many mixed LnMOFs have been fabricated for temperature measurement based on similar luminescent behavior [122].

**Figure 12:**
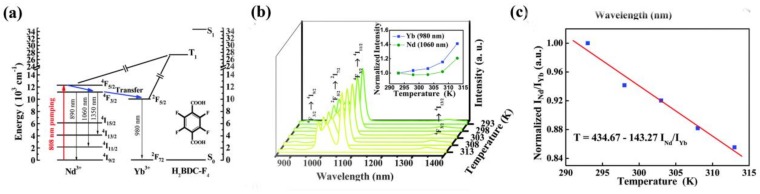
(**a**) Schematic representation of energy processes in Nd_0.577_Yb_0.423_BDC-F_4_. (**b**) Emission spectra of Nd_0.577_Yb_0.423_BDC-F_4_ in the range of 293–313 K excited at 808 nm; inset: temperature dependence of the normalized intensity of the corresponding transitions. (**c**) Temperature-dependent intensity ratio of Nd^3+^ (1060 nm) to Yb^3+^ (980 nm) and the fitted curve for Nd_0.577_Yb_0.423_BDC-F_4_. Reproduced with permission from [123]. Copyright 2015 The Royal Society of Chemistry.

In 2015, another mixed LnMOF (Nd_0.577_Yb_0.423_)_2_(BDC-F_4_)_3_(DMF)(H_2_O)⋅DMF (H_2_BDC-F_4_ = 2,3,5,6-tetrafluoro-1,4-benzenedicarboxylate) with typical NIR emission for temperature sensing was designed by Qian and coworkers (Figure 12a) [123]. NIR emission can enter the biological system because of its relatively small adsorption and scattering. Thus, such NIR temperature thermometers have great potential for monitoring temperature in biological systems. The intensity ratio of Nd^3+^ at 1060 nm and Yb^3+^ at 980 nm is linearly related to temperatures in the physiological range (293–313 K) with a relative sensitivity of 0.816% K^−1^ at 313 K (Figure 12b,c), suitable for use in biomedical diagnosis.

**Figure 13:**
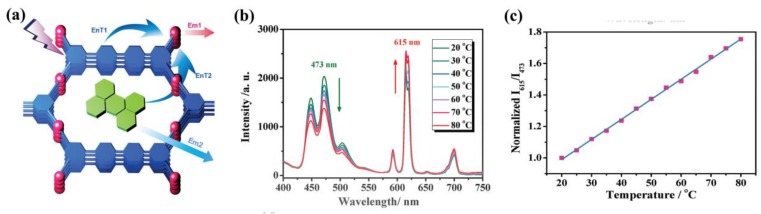
(**a**) Design of dual-emitting ZJU-88⊃perylene composite (EnT: energy transfer, Em: emission); (**b**) Emission spectra of ZJU-88⊃perylene recorded from 20 to 80 °C, excited at 388 nm; (**c**) Temperature-dependent intensity ratio of Eu^3+^ (615 nm) to perylene (473 nm) and the fitted curve for ZJU-88⊃perylene. Reproduced with permission from [124]. Copyright 2015 WILEY-VCH.

More recently, Qian and coworkers suggested that ratiometric temperature sensors can be achieved by the MOF⊃luminescent guest species composite method because of the energy transfer between luminescent guest species and Ln^3+^ ions [124]. The ZJU-88⊃perylene composite (ZJU⊃88 = [Eu_2_ (QPTCA)(NO_3_)_2_(DMF)_4_]·(CH_3_CH_2_OH)_3_, QPTCA = 1,1′:4′,1″:4″,1′″-quaterphenyl-3,3′″,5,5′″-tetracarboxylic acid)) was designed as a dual-emitting thermometer with high sensitivity (1.28% °C^−1^ at 20 °C) (Figure 13a). Further results showed that the ZJU-88⊃perylene had good stability, an outstanding linear relationship, and excellent biocompatibility under simulated physiological conditions (Figure 13b,c), all indicating its potential use as a luminescent thermometer in biological applications. 

### 3.8. LnMOFs for Biosensing

Nitrofurans are a type of extensively used veterinary antibiotics effective for the treatment of protozoan and bacterial infections in human beings. It is, however, still urgently needed, as well as very challenging to develop a rapid and effective approach to detect nitrofuran antibiotics (NFAs) [125]. Yang and coworkers fabricated a Eu-BCA ({[Eu_2_(BCA)_3_(H_2_O)(DMF)_3_]·0.5DMF·H_2_O}_n_, BCA = 2,2′-biquinoline-4,4′-dicarboxylate) thin-film sensor for NFAs by coating a cost-effective stainless-steel wire mesh using the Co_3_O_4_ nano-anchor fixation approach. The Eu-BCA thin-film sensor shows significant quenching effect for NFAs owing to the synergistic effect of electron-transfer and the inner-filter effect. It furthermore shows high selectivity and sensitivity to NFAs with detection limits of 0.21 and 0.16 mm for nitrofurantoin (NFT) and nitrofurazone (NFZ), respectively. NFAs were also successfully detected in real samples, indicating the potential of this Eu-BCA thin-film for biosensing [126].

**Figure 14:**
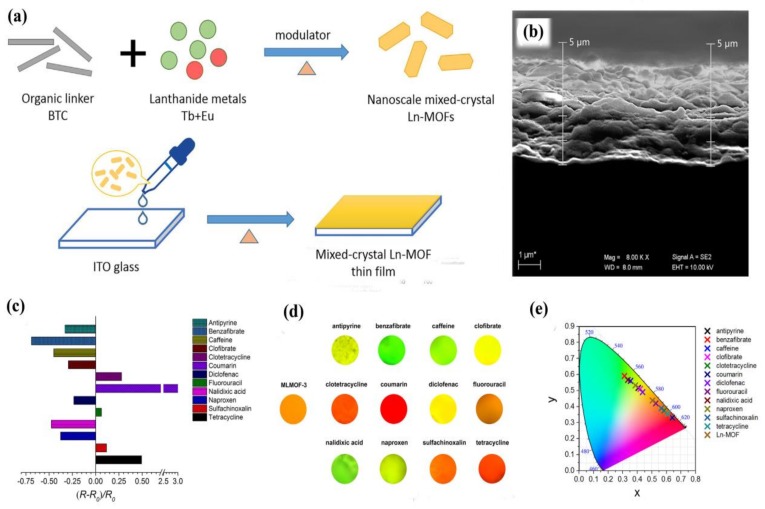
(**a**) Preparation process of mixed-crystal LnMOF thin film; (**b**) SEM image of cross-section of the film; (**c**) the emission intensity ratio changes; (**d**) the optical photographs; and (**e**) CIE chromaticity coordinates of the LnMOF thin film in the presence of different analytes. (20 mL, 10^−4^ M). Reproduced with permission from [127]. Copyright 2017 Elsevier B.V.

Another pharmaceutical sensor was designed by Wang and coworkers based on a luminescent mixed-crystal LnMOF (MLMOF-3 = Eu_0.1_Tb_0.9_-BTC) thin film [127]. The uniform and continuous thin film was prepared by coating the monodisperse nanoscale MLMOF-3 on indium–tin–oxide (ITO) glass (Figure 14a,b). The luminescence intensity ratios of Eu^3+^ at 619 nm to Tb^3+^ at 547 nm of the MLMOF-3 film were used to calculate the intensity ratio change by (R–R_0_)/R_0_, where R_0_ is the initial intensity ratio without the analyte, and R is the intensity ratio upon the addition of the analyte (Figure 14c). The luminescence intensity depended significantly on several pharmaceutical molecules (such as antipyrine, benzafibrate, caffeine, clofibrate, clotetracycline, coumarin, diclofenac, fluorouracil, nalidixic acid, naproxen, sulfachinoxalin, and tetracycline) Moreover, the MLMOF-3 thin film shows different guest-dependent colors that can intuitively be distinguished by the naked eye (Figure 14d,e). The authors presumed that the different functional groups and structures of these pharmaceutical molecules may not only modulate the antenna effect between organic linkers and Ln^3+^ ions but also affect energy transfer between Tb^3+^ and Eu^3+^, causing the different luminescent changes in the MLMOF-3 thin film. These results demonstrate that the mixed LnMOF film can be used as luminescence sensors for different pharmaceutical molecules.

Yan and coworkers were the first to design a diagnosis platform for vinyl chloride carcinogen based on a 3d–4f–4d heterometallic MOF (Eu^3+^/Cu^2+^-Zr_6_O_4_(OH)_4_(O_2_C-C_6_H_2_-CO_2_(CO_2_H)_2_)_6_·*x*H_2_O) [128]. The nanoprobe exhibits high selectivity to thiodiglycolic acid (TDGA) with a luminescence enhancement of about 27.5-fold, the main metabolite of vinyl chloride monomer (VCM) in human urine. It further shows a fast response to TDGA within 4 min and impressive sensitivity with a detection limit of 89 ng·mL^−1^ without interference of other coexisting species in urine. Such excellent sensing performance enables it to monitor TDGA levels in human urine. Furthermore, a portable urine dipstick based on the sensor has been developed to conveniently evaluate individual’s intoxication degree of VCM. 

## 4. Conclusions and Outlook

This comprehensive review covers the recent research progress on luminescent lanthanide MOFs and their applications in sensing cations, anions, small molecules, nitroaromatic explosives, gases, vapors, pH, temperature, and biomolecules. The sensing functionality of LnMOF probes is based on their luminescence changes in response to different analytes, all recognizable by means of spectrofluorometry or the naked eye. Most of the luminescent LnMOF sensors operate by a turn-off mechanism when detecting electron acceptors in which luminescence is quenched through both the electron and the energy transfer between LnMOF sensors and analytes. However, the turn-on detection mode with higher sensitivity and lower detection limits has also been implemented in luminescence-based LnMOF sensors resulting in luminescence enhancement or wavelength shifts. Furthermore, rational incorporation of the functional sites (e.g., Lewis acidic or basic sites and open metal sites) on the pores of the LnMOFs has made them very promising sensors to detect target compounds. Moreover, the ratiometric sensing approach has easily been achieved by embedding multi-luminescent motifs onto the frameworks, which can overcome the main drawbacks of the intensity-based measurements with only one transition. 

Although the sensing behavior of LnMOFs has been studied comprehensively, some problems remain. While many investigations have shown excellent results for sensing hazardous materials, fast detection of nitroexplosives with a handheld device in public places, such as the airport and railway station, stays challenging. Furthermore, nanoscale luminescent MOFs with controllable size and morphology are very promising in applications for sensing in living cells. More efforts should be devoted to integrating different functionalities such as cellular sensing and imaging and molecular targeting, as well as drug delivery for practical applications in theranostic nanomedicine. Moreover, in-depth studies on the relationships between structure and luminescent behavior must be conducted using theoretical methods. In addition, the stabilities of recycling, material cost, and portability for practical applications need further improvement. With constant efforts being made to handle these challenges, we believe that the LnMOFs definitely hold a bright future in the field of luminescence sensing.

## Abbreviations

Ad	adenine
BCA	2,2′-biquinoline-4,4′-dicarboxylate
H_2_BDC	1,4-benzenedicarboxylic acid
H_2_BDC-F_4_	2,3,5,6-tetrafluoro-1,4-benzenedicarboxylate
bipy	4,4′-bipyridine
H_2_BPDC	2,2′-bipyridine-3,3′-dicarboxylic acid
BPT	biphenyl-3,4′,5-tricarboxylate
bpydc	2,2′-bipyridine-5,5′-dicarboxylic acid
H_3_BTB	1,3,5-benzenetribenzoate
BTC	benzene-1,3,5- tricarboxylate
H_4_btec	pyromellitic acid
H_4_BTMIPA	5,5′-methylenebis(2,4,6-trimethylisophthalic acid)
CB	conduction band
CDs	carbon dots
HCHO	formaldehyde
CPNPs	coordination polymer nanoparticles
H_2_CPOC	5-(4′-carboxylphenoxy) nicotinic acid
DMA	dimethylacetamide
DMBDC	2,5-dimethoxy-1,4-benzenedicarboxylate
DMF	N′N-dimethylformamide
DPA	dipicolinic acid
H_2_FDC	9,9-dimethyl-2,7-fluorenedicarboxylic acid
FBPT	2′-fluoro-biphenyl-3,4′,5-tricarboxylate
FTIR	Fourier-transform infrared spectroscopy
fum	fumarate
ICT	intramolecular-charge-transfer
H_2_ipbpBr	1-(3,5-dicarboxyphenyl)-4,4′-bipyridinium bromide
ITO	indium–tin–oxide
H_2_L_1_	2,5-di(pyridin-4-yl)terephthalic acid
H_2_L_2_**^−^**	3, 5-dicarboxy-phenol anion ligand
L_3_	4,4′-dicarboxylate-2,2′-dipyridine anion
H_3_L_4_	p-terphenyl-3,4″,5-tricarboxylic acid
H_3_L_5_	4-(2-carboxyphenoxy)benzene-1,3-dioic acid
H_2_L_6_	5-(4*H*-1,2,4-triazol-4-yl)benzene-1,3-dicarboxylic acid
L_7_	2′,5′-bis(methoxymethyl)-[1,1′:4′,1″-terphenyl]-4,4″-dicarboxylate
LMCT	ligand-to-metal charge transfer
LnMOFs	lanthanide metal–organic frameworks
LUMOs	lowest unoccupied molecular orbitals
Mg-MOF	{[Mg_3_(ndc)_2.5_(HCO_2_)_2_(H_2_O)][NH_2_Me_2_]⋅2H_2_O⋅DMF}
MIL-61	Ga(OH)(btec)·0.5H_2_O
MLCT	metal-to-ligand charge transfer
MOFs	Metal–organic frameworks
mpca	2-pyrazine-5-methyl-carboxylate
H_4_mtb	4-[tris(4-carboxyphenyl)methyl]benzoic acid
MTT	3-(4,5-dimethylthiazol-2-yl)-2,5-diphenyl-tetrazolium bromide
H_2_NDC	2,6-naphthalenedicarboxylate
NB	nitrobenzene
1,4-ndc	1,4-naphthalenedicarboxylate
NFAs	nitrofuran antibiotics
NFT	nitrofurantoin
NFZ	nitrofurazone
2,4-NP	2,4-dinitrophenol
3-NP	3-nitrophenol
4-NP	4-nitrophenol
N-GQDs	N atom-doped graphene quantum dots
m-NT	m-nitrotoluene
o-NT	o-nitrotoluene
NTA	nitrilotriacetate
OBA	4,4′-oxybis(benzoate)
ODA	oxydiacetic acid
ox	oxalate
HPAN	hydrolyzed polyacrylonitrile
PDC	pyridine-3,5-dicarboxylate
PET	photoinduced electron transfer
Phen	1,10-phenanthroline
QPTCA	1,1′:4′,1″:4″,1′″-quaterphenyl-3,3′″,5,5′″-tetracarboxylic acid
H_2_S	hydrogen sulfide
tctpH_3_	tris(p-carboxylato)triphenylphosphine
TDGA	thiodiglycolic acid
TNP	2,4,6-trinitrophenol
TNT	2,4,6-trinitrotoluene
VCM	vinyl chloride monomer
ZIF-8	zeolitic imidazolate framework-8
